# On the reliability of a simple method for scoring phenotypes to estimate heritability: A case study with pupal color in *Heliconius erato phyllis*, Fabricius 1775 (Lepidoptera, Nymphalidae)

**DOI:** 10.1590/S1415-47572009005000005

**Published:** 2009-01-10

**Authors:** Adriano Andrejew Ferreira, Luiz Carlos Kucharski, Aldo Mellender de Araújo

**Affiliations:** 1 Departamento de Genética, Instituto de Biociências, Universidade Federal do Rio Grande do Sul, Porto Alegre, RSBrazil; 2 Departamento de Fisiologia, Universidade Federal do Rio Grande do Sul, Porto Alegre, RSBrazil

**Keywords:** allocation-priority hypothesis, butterfly, optical density, pupal melanization, qualitative and quantitative methods

## Abstract

In this paper, two methods for assessing the degree of melanization of pupal exuviae from the butterfly *Heliconius erato phyllis*, Fabricius 1775 (Lepidoptera, Nymphalidae, Heliconiini) are compared. In the first method, which was qualitative, the exuviae were classified by scoring the degree of melanization, whereas in the second method, which was quantitative, the exuviae were classified by optical density followed by analysis with appropriate software. The heritability (h^2^) of the degree of melanization was estimated by regression and analysis of variance. The estimates of h ^2^ were similar with both methods, indicating that the qualitative method could be particularly suitable for field work. The low estimates obtained for heritability may have resulted from the small sample size (*n* = 7-18 broods, including the parents) or from the allocation-priority hypothesis in which pupal color would be a lower priority trait compared to morphological traits and adequate larval development.

## Introduction

Butterflies have traditionally been used to solve problems in ecological genetics and evolution (see, for example, Kapan, 2001; Ruszczyk *et al.*, 2004; Kronforst *et al.*, 2006; Mavárez *et al.*, 2006; Cardoso and Gilbert, 2007). In his well-known and influential book, Ford (1975, p. 9) emphasized that “among many other forms, the Lepidoptera have up to now been extensively used in ecological genetics. This is partly, but by no means wholly, accidental. [...] their wing-patterns do provide exceptional opportunities for detecting phenotypic variation; and it will be noticed that, in general, they possess a large number of the desirable qualities listed above.” The complex life-cycle of butterflies, during which selection pressures may differentially influence immature and adult forms, also makes these insects useful models (Benson, 1971; Mega and Araújo, 2008). Another well-explored aspect of butterfly development is the variation in pupal color, which can be polymorphic with a strong environmental influence (Smith *et al.*, 1988; Hazel *et al.*, 1998) or may show phenotypes with continuous variation (Starnecker and Hazel, 1999).

We recently described pupal melanization in *Heliconius erato phyllis*, Fabricius 1775 (Nymphalidae, Heliconiinae) and the role played by genetic and environmental factors in the expression of this phenotype (Ferreira *et al.*, 2006). Twenty-eight inbred and non-inbred broods were subjected to different treatments designed to distinguish between genetic and environmental influences. For convenience, the pupal phenotypes were scored in discrete units, with 2 as the lightest color and 5 as the darkest.

In the present work, we compared the suitability of two methods for estimating the heritability of pupal color in *H. erato phyllis*. The samples used in the previous study and additional broods were scored as described above. The pupae were also measured with a continuous scale based on optical density. Despite the simplicity of the former method in which only a few scores based on visual inspection were used the heritability estimates were as good as those obtained by using a more sophisticated method.

## Material and Methods

*Heliconius erato* is a Neotropical butterfly with a remarkable variety of forms and local races that have different wing colors. Local races of *H. erato*, together with those of *Heliconius melpomene*, Linnaeus (Nymphalidae) represent one of the most spectacular examples of mimicry (Sheppard *et al.*, 1985). The so-called “East Brazilian Race”, *Heliconius erato phyllis*, has been the subject of numerous studies in Brazil over the past 20 years (*e.g.*, Saalfeld and Araújo, 1981; Pansera and Araújo, 1983; Périco and Araújo, 1991; Silva and Araújo, 1994; Ramos and Freitas, 1999; Rodrigues and Moreira, 1999). Adults of *H. erato phyllis* adapt easily to life in insectaries, where all stages of the life cycle can be studied.

### Rearing immature and adult forms of *H. erato phyllis*

All pre-adult development of *H. erato phyllis* occurred under continuous light at 25 ± 1 °C in translucent plastic pots (8.5 cm high and 7.5 cm wide) with a white lid and a bottom covered with white soft paper. Each pot housed only one caterpillar that was daily fed with *Passiflora suberosa*, Linnaeus or *Passiflora misera*, Humboldt (Passifloraceae).

### Colour analysis of exuviae

After emergence of the adults, the resulting pupal exuviae were compressed between a translucent slide and the extremities were fixed with a label that contained information on the rearing conditions, degree of pupal melanization, sex of the adult and other phenotypic indicators. The extent of melanization of the pupal exuviae was assessed by visual inspection (qualitative analysis) or by measuring the optical density (quantitative analysis).

The optical densities of pupal exuviae were measured with an Imagemaster VDS photo-documentation system (GE Healthcare, Piscataway, NJ, USA). This equipment was set to operate by transmittance with a yellow filter, aperture f/8, focus 2, and zoom 12. Potential deviations that could be produced by each measurement were corrected by using a grey scale that consisted of eight small rectangles colored from 30% to 100% black and printed on a slide. Four slides with pupal exuviae and the slide with a grey scale were measured each time. The area chosen in each exuvia corresponded to the butterfly wing. The same slide with the grey scale was used for all measurements of pupal exuviae. The images were stored and analysed using the software Image J 1.34 m (Rasband, 2005) at a resolution of 43.2 pixels/cm. The *polygon* tool was used to draw the figure to be measured in the butterfly wing area and, for each exuvia, the software calculated the mean number of pixels for the polygon (pixel scale ranged from 0 for black to 255 for white). This mean value was referred to as the *raw exuvia score* (RES).

To correct for possible deviations in the image captured during photo-documentation *polygon* tool and the rectangle corresponding to 60% black in the grey scale were used to draw a figure equivalent to that of the exuviae to be analysed by the software Image J 1.34m. The resulting value was referred to as the *control by capture* (CC). After all of the slides had been measured (n = 648), the mean value (referred to as the *mean of the controls* or MC) was calculated and a *correction ratio* (CR) was obtained by dividing CC by MC. The RES/CR ratio for each exuvia was then calculated and this new ratio was used to estimate heritability.

The repeatability of the two methods (Lessels and Boag, 1987) was assessed by extracting a random sample of 65 exuviae that was proportional to brood size. Each exuvia was scored (first method) and measured (second method) three times after reshuffling the exuviae. The repeatability for the qualitative and quantitative methods was 0.8840 ± 0.0224 and 0.9975 ± 0.0005, respectively.

### Heritability of pupal color

Estimates of heritability (h^2^) for the qualitative and quantitative methods were calculated by conventional regression and analysis of variance. The procedures applied to the qualitative method were also used with the quantitative method (for details, see Ferreira *et al.*, 2006). The sample sizes used here were slightly different from our previous report since we tried to use an equal number of exuviae in both methods. Brood size varied from 17 to 51, with an average of 36 individuals.

## Results

Table 1 shows the heritability estimates calculated by regression and analysis of variance for qualitative scoring of pupal color. The estimates were virtually the same as those reported by Ferreira *et al.* (2006). These findings indicate that, regardless of the method used to estimate heritability (regression or analysis of variance), the h^2^ value was low (not significantly different from zero, except for two values indicated in Table 1). In addition, when the female parental score was considered as the independent variable in regression, the estimates of heritability were lower than the values normally obtained (negative h^2^ values in Table 1). When only the female parental scores were considered, the number of broods was higher than for male parents, partly because in some crosses we were unable to identify which male copulated with a given female. However, since *H. erato phyllis* females are monogamous, this did not influence the heritability estimates. Table 2 shows the heritabilities estimated by the optical density method. Although this method used a continuous scale for classifying the exuviae phenotypes, the h^2^ values were of the same order of magnitude as those obtained by the qualitative method. Again, none of the values was significantly different from zero, although the results for male parents (second column, Table 2) were somewhat higher than the corresponding values in the qualitative method. The results obtained when the mid-parental value was used as the independent variable in regression analysis were similar to those obtained by analysis of variance, and the h^2^ values remained low and negative when the female values were used as the independent variable.

## Discussion

Pupae of the butterfly *H. erato phyllis* vary in color from light to dark, both in the field and in captivity. Variation is the first condition for natural selection. However, in order to evolve, phenotypes such as pupal color must be inherited, which is why we estimated the heritability of this trait; the possible role of natural selection in this phenomenon is now being studied by our group. Both of the methods used to score pupal color in this study showed that this trait had low heritability, a finding that may be of considerable evolutionary importance.

Of the two methods used here, the qualitative method, which was based on a scale of increasing melanization from 2 to 5, was established after simultaneous visual inspection of the exuviae by two of the authors (AAF and AMA). The quantitative method, which was more demanding, was based on optical density and resulted in a continuous scale of melanization. Both of the methods yielded almost identical estimates of heritability. Since it is easier to score pupal phenotypes by visual inspection, particularly during field work, and the repeatability of the qualitative method was as high as that of the quantititave method, we propose that the former be accepted as a reliable method.

The finding that the estimates of heritability for pupal melanization in *H. erato phyllis* were almost identical with both methods raises the question as to why a phenotype, which is presumably related to survival, should have low heritability. A further complication is that pupal color in this species is strongly influenced by the environment (Ferreira *et al.*, 2006). There are at least two explanations for these results. First, when heritability estimates are calculated by offspring-parent regression significant results can often only be obtained with a large number of parents and offspring (> 100) that provide a small standard error, as pointed out by Falconer (1989). Second, the failure to obtain a high heritability estimate depends on the trait being measured. Glazier (2002) discussed a very interesting possibility, which he called the allocation-priority hypothesis. In this hypothesis, low priority traits in the resource-allocation system are more affected by environmental variation, thereby reducing their heritability.

A priority rule in the case of pupation is its appropriate development and morphology, with color being secondary. Pupal melanization should be viewed as a means of enhancing the chances of survival in the presence of visually-oriented predators. We have examined the effects of different degrees of melanization on the survival of pupae in nature and our results strongly support the hypothesis that color is not an important variable in pupal mortality since their main predators in the area studied were ants (A.A. Ferreira and A.M. Araújo, unpublished observations). Although the allocation-priority hypothesis is extremely interesting, it requires careful testing under field and laboratory conditions.

## Figures and Tables

**Table 1:** Heritability estimates (h^2^) calculated by regression and analysis of variance (ANOVA) (inbred broods are not included) based on a qualitative melanization score.

Sibship	Regression (parent *vs.* offspring)			ANOVA
	Midparental value	Male parent	Female parent	
Mean	0.40 ± 0.21 (7)	0.44 ± 0.28 (7)	-0.11 ± 0.23 (12)	0.31 ± 0.11 (18)
Males	0.36 ± 0.15 (7)^1^	0.43 ± 0.20 (7)^2^	-0.20 ± 0.24 (12)	0.34 ± 0.13 (18)
Females	0.47 ± 0.32 (7)	0.49 ± 0.41 (7)	-0.01 ± 0.26 (12)	0.32 ± 0.12 (18)

The values are the mean + s.e. of the total number of broods indicated in parentheses. ^1^p = 0.064, ^2^p = 0.083.

**Table 2:** Heritability estimates (h^2^) calculated by regression and analysis of variance (inbred broods are not included) based on a continuous (quantitative) melanization scale.

Sibship	Regression (parent *vs.* offspring)			ANOVA
	Mid-parental value	Male parent	Female parent	
Mean	0.31 ± 0.25 (7)	0.61 ± 0.30 (7)	-0.04 ± 0.20 (12)	0.30 ± 0.10 (18)
Males	0.30 ± 0.18 (7)	0.42 ± 0.27 (7)	-0.03 ± 0.19 (12)	0.29 ± 0.12 (18)
Females	0.34 ± 0.36 (7)	0.84 ± 0.42 (7)	-0.06 ± 0.27 (12)	0.32 ± 0.12 (18)

The values are the mean ± s.e. of the total number of broods indicated in parentheses.
